# Socially interactive agents for robotic neurorehabilitation training: conceptualization and proof-of-concept study

**DOI:** 10.3389/frai.2024.1441955

**Published:** 2024-11-28

**Authors:** Rhythm Arora, Pooja Prajod, Matteo Lavit Nicora, Daniele Panzeri, Giovanni Tauro, Rocco Vertechy, Matteo Malosio, Elisabeth André, Patrick Gebhard

**Affiliations:** ^1^German Research Center for Artificial Intelligence, Saarbrücken, Germany; ^2^Human-Centered Artificial Intelligence, Augsburg University, Augsburg, Germany; ^3^National Research Council of Italy, Lecco, Italy; ^4^Industrial Engineering Department, University of Bologna, Bologna, Italy; ^5^Scientific Institute IRCCS E. Medea, Bosisio Parini, Lecco, Italy

**Keywords:** social agent, virtual coach, robotic neurorehabilitation, behavior adaption, human-robot interaction, affective computing

## Abstract

**Introduction:**

Individuals with diverse motor abilities often benefit from intensive and specialized rehabilitation therapies aimed at enhancing their functional recovery. Nevertheless, the challenge lies in the restricted availability of neurorehabilitation professionals, hindering the effective delivery of the necessary level of care. Robotic devices hold great potential in reducing the dependence on medical personnel during therapy but, at the same time, they generally lack the crucial human interaction and motivation that traditional in-person sessions provide.

**Methods:**

To bridge this gap, we introduce an AI-based system aimed at delivering personalized, out-of-hospital assistance during neurorehabilitation training. This system includes a rehabilitation training device, affective signal classification models, training exercises, and a socially interactive agent as the user interface. With the assistance of a professional, the envisioned system is designed to be tailored to accommodate the unique rehabilitation requirements of an individual patient. Conceptually, after a preliminary setup and instruction phase, the patient is equipped to continue their rehabilitation regimen autonomously in the comfort of their home, facilitated by a socially interactive agent functioning as a virtual coaching assistant. Our approach involves the integration of an interactive socially-aware virtual agent into a neurorehabilitation robotic framework, with the primary objective of recreating the social aspects inherent to in-person rehabilitation sessions. We also conducted a feasibility study to test the framework with healthy patients.

**Results and discussion:**

The results of our preliminary investigation indicate that participants demonstrated a propensity to adapt to the system. Notably, the presence of the interactive agent during the proposed exercises did not act as a source of distraction; instead, it positively impacted users' engagement.

## 1 Introduction

Neurorehabilitation is a widely used medical practice that aims to aid recovery from a nervous system injury. Its purpose is to maximize and maintain the patient's motor control while trying to restore motor functions in people with neurological impairments. Given the constant growth and aging of the world population, the number of patients affected by neuromotor disorders that seek the attention of professionals for their rehabilitation therapy is constantly increasing (Crocker et al., 2013). However, due to a lack of medical personnel, it is impossible to provide the intense training that would be needed for an effective recovery of the patient's capabilities, therefore hindering the actual outcomes of the treatment (Teasell et al., 2005).

This situation is both harmful for the patients and constitutes a relevant burden on society and the healthcare system (Wynford-Thomas and Robertson, 2017). To address this issue, robot-assisted training has been widely investigated as an effective neurorehabilitation approach that helps augment physical therapy and facilitates motor recovery. According to literature, such approaches can help therapists save time and energy while providing patients with a tool capable of assisting the execution of accurate and repetitive moments in high-intensity training sessions (Kwakkel et al., 2008; Zhang et al., 2017; Qassim and Wan Hasan, 2020). The current situation sees a limited number of this kind of devices, already installed in rehabilitation clinics, hindering their potential as they have to be scheduled over a large number of patients (Maciejasz et al., 2014; Stein, 2012). However, forecasts show that a relevant diffusion of this technology is taking place meaning that, in the near future, we will see an exponentially rising number of the installations of this technology (Morone et al., 2023). Moreover, most of the devices currently available are bulky and expensive but, thanks to the push for telemedicine and telerehabilitation, a new generation of rehabilitation robots is making its way into the market (Washabaugh et al., 2018; Molaei et al., 2022; Mayetin and Kucuk, 2022; Tseng et al., 2024). These affordable and portable solutions would allow for the capillary diffusion of the technology, out of the clinics and directly at home for the patients to use. The application of rehabilitation robots in domestic environments would represent a plausible solution to the lack of treatment intensity that patients are experiencing nowadays. In fact, a system capable of assisting the patient in performing the necessary repetitive motions would relieve a lot of the pressure that is acting on the clinical structures, since the physical presence of medical personnel would be required only for sporadic interventions. However, a crucial issue for rehabilitation training is user engagement and motivation (Blank et al., 2014), which may be lacking if the rehabilitation system is used without a human medical coach. Since the effectiveness of the treatment has been proven to be related to the patient's level of engagement (Turner-Stokes et al., 2015), it is important for the envisioned system not only to be able to physically assist the patients but also to understand their affective state and react accordingly. Hence, we believe that introducing a socially-aware interactive virtual agent could represent a promising solution to recreate the social aspects of in-person rehabilitation sessions. In this scenario, having a socially interactive agent can help support the patients' engagement and motivation in a flexible and personalized way. It is important to state that professional physiotherapists would still play a fundamental role in this home-based robotic treatment. In fact, the system is envisioned to be used by patients at home only after a training phase. During this phase, the system is required to learn directly from the experience of professional physiotherapists how to respond to the needs of the specific patient (Lequerica et al., 2009). In particular, the system should be able to understand both the patient's residual physical capabilities, in order to provide a properly tuned level of assistance, and the behavioral patterns that should be elicited by the virtual coach in order to keep the patient engaged in the exercise. Moreover, insights from the field of social robotics suggest that the enhancement of a rehabilitation system through affective and social signal processing can augment the personalization of the system and further facilitate neuroplasticity (Nahum et al., 2013). Therefore, a neurorehabilitation training system capable of modeling the patient's state and tuning its behavior depending on both the measured performance deviation index and the inferred mental and physical state could improve the user's engagement and the outcome of the therapy. The performance deviation index, which is inversely proportional to the user's performance, represents the deviation of the actual path followed by the participant from the ideal path.

**Figure 1:**
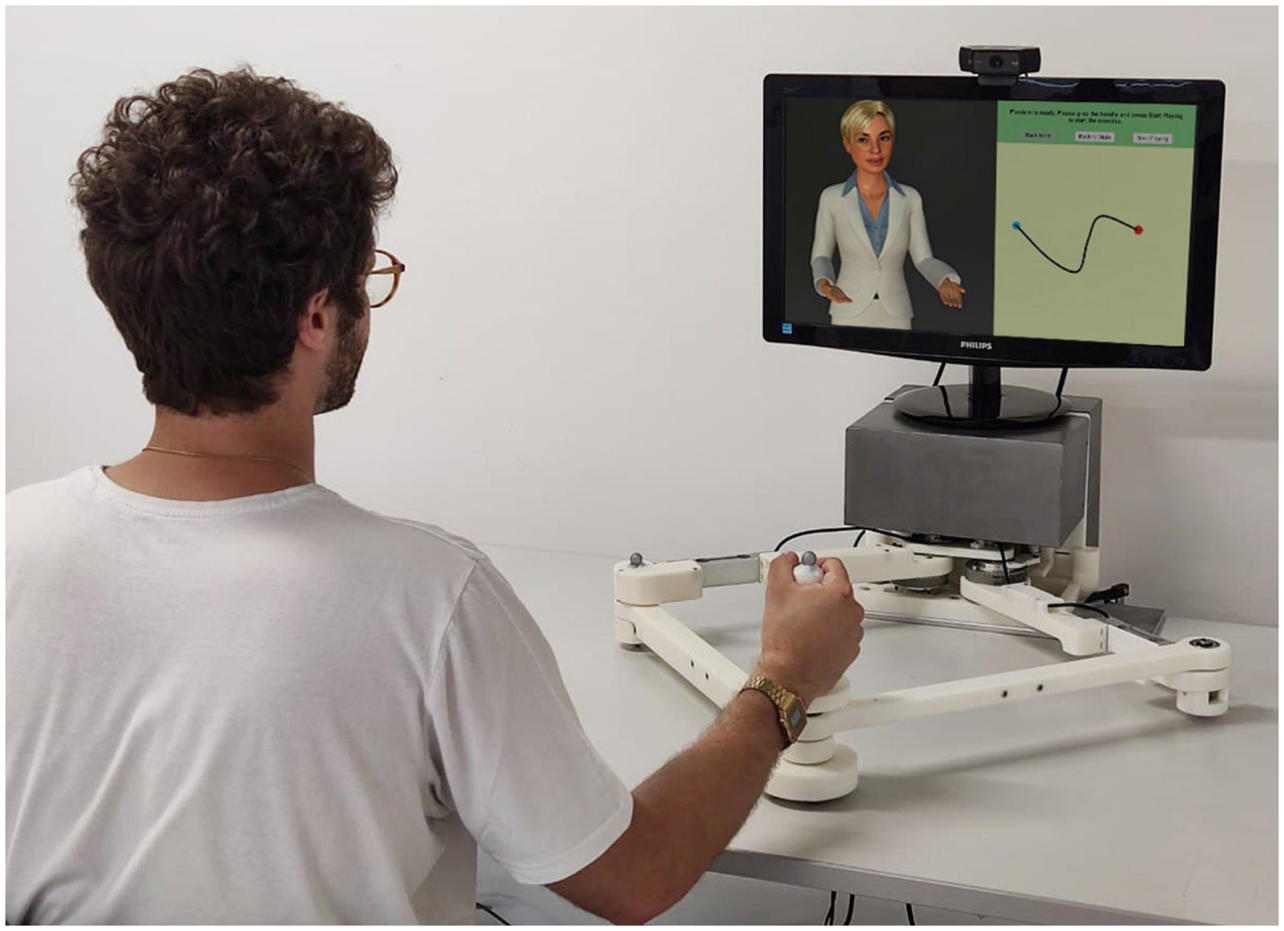
Empathetic Neurorehabilitation Trainer concept.

This paper investigates the feasibility of the Empathetic Neurorehabilitation Trainer depicted in Figure 1, a technology-based upper-limb neurorehabilitation system equipped with social interaction capabilities and composed of (1) a planar rehabilitation device for physical assistance and (2) a socially interactive virtual agent in the role of a supportive coaching assistant. In this context, the physiological and behavioral signals of the user are collected, analyzed, and elaborated into attentiveness, stress, and pain information, which are then exploited to tune the rehabilitation session coherently. Proving the feasibility of the proposed approach represents a first step in the direction of smart and scalable robot rehabilitation systems which would be beneficial in closing the gap between the need of intense training and the lack of available personnel.

The paper is structured as follows: Section 2 provides a complete overview of the background knowledge and related works on which the rest of the study is based on. Section 3 presents the materials and methods used to build the proof-of-concept system tested in Section 4. Finally, Section 5 is dedicated to the discussion of the collected outcomes before the conclusions drawn in Section 6.

## 2 Background and related works

Before going straight into the description of the envisioned Empatic Neurorehabilitation Trainer, an overview of the background knowledge gathered and analyzed as a basis for the conceptualization of the system is reported.

### 2.1 Neurorehabilitation best practices

In order to gather precious insights on the strategies and struggles that professional neurorehabilitation therapists experience during their daily practice, we decided to perform a series of interviews online through Microsoft Teams as free open discussions. Overall, we were able to collect the point of view of 15 therapists spread over the Italian territory and here a summary of the obtained insights is reported.

The constantly growing employment of technological devices in neurorehabilitation therapy can be explained by the introduced ease in reaching a significant number of movement repetitions in a specific body district (body parts grouped by functionality) (Bonney et al., 2017) and in obtaining higher patient engagement, fundamental aspects in the motor rehabilitation process. In this regard, one of the interviewees said (translated from Italian): “I like to use technological solutions for the intensity they can provide and for the possibility to perform rehabilitation also in very extreme cases.” However, even when relying on robotic solutions, one must be able to balance movement repetition, often leading to boredom, with variability and incentive to enhance the patient's attention and commitment.

During every therapy session, a therapist assesses the actual level of attention, commitment, engagement, stress, and pain currently experienced by the patient to deliver the correct amount of exercise and to avoid the risk of too easy or too difficult tasks that may lead to a decay in interest or even a feeling of frustration (Flores et al., 2008; King et al., 2010; Zimmerli et al., 2013). In order to reach this goal, the therapist relies on the activity scores and the patient's behavior. For example, if the patient cannot achieve a particular performance deviation index, the selected activity is likely too difficult. On the other hand, if the patient can perform the exercise but, after some time, becomes very talkative and less performing, it is likely that a decay in interest is occurring. In such cases, the therapist must give feedback and, if needed, support when the activities are too difficult or change the exercise when the attention starts to decrease. Furthermore, considering patients affected by neurological impairments, attention problems are frequent. Recording the period to which the attention lasts can be valuable information to provide a correct dosage of exercise. Lastly, the management of neurological disorders can be considerably different between adults and children. In both cases, understanding when a pause or a change of exercise is necessary is crucial. In this regard, one of the interviewees said (translated from Italian): “The currently available robotic devices often require the patient to adapt to them instead of the opposite. An automatic tuning of the exercise duration and difficulty to the needs of the patients would be game-changing.” Moreover, considering adults, one can count on their responsibility to train toward an improvement, even if the activity could lead to boredom. On the other hand, children may not behave in the same way and, to augment their engagement, it is crucial to introduce gaming aspects to the exercise.

### 2.2 Control logics

The first goal of a neurorehabilitation robot control algorithm is the ability to elicit neuroplasticity and enhance the patient's motor recovery. To make this possible, it is crucial that the assistance provided by the device is not too low in order to allow the patient to complete the task and to avoid frustration, but also not too high, thereby ensuring that the patient actively participates in the task with no risk of slacking (Erdogan and Patoglu, 2012). Also, it is important that the device does not perfectly correct the motion initiated by the user. In fact, a certain amount of error has been proved to be useful in stimulating neuroplasticity given that the patient has to put focus and effort on the task in order to correct the motion autonomously as much as possible (Takagi et al., 2018). Thus, the capability of a robot to actively and automatically adapt the level of assistance according to the skills and the performances of the patient is one of its most important features (Marchal-Crespo and Reinkensmeyer, 2009; Meng et al., 2015).

Furthermore, the level of provided assistance should not be defined only on the basis of the kinematic performances of the patient. In this regard, the evaluation of social, physiological and psychological aspects provides a more fine-grained assessment of the patient's state, useful to achieve a better tuning of the behavior of the system (Novak et al., 2011; Malosio et al., 2016). For instance, as mentioned in Section 2.1, a patient that starts feeling bored will be less engaged on the task with the risk of reducing the effectiveness of the exercise. A system capable of detecting this state could, instead, render the task more challenging, for instance by reducing the level of provided assistance, to bring the patient's focus back on the exercise.

### 2.3 Affective signal interpretation

During the training sessions, the affective signals collected from the patients can be used to infer useful information about their experience. Home-based healthcare systems frequently leverage a diverse range of affective signals (Majumder et al., 2017; Philip et al., 2021; Wang et al., 2021). In this section, we provide a brief description of three affective states integral to neurorehabilitation, along with an overview of typical modalities utilized for inferring these states.

- **Attention**: Motivation and attention serve as crucial modulators of neuroplasticity, influencing the outcomes of rehabilitation therapy (Cramer et al., 2011). Distractions, stemming from factors like boredom or lack of motivation, can disrupt the user's engagement during training sessions. Hence, the user's attention level becomes a pivotal input for the agent's motivational strategy in neurorehabilitation. While previous studies in various domains have demonstrated the prediction of attention through physiological signals such as EEG (Acı et al., 2019; Souza and Naves, 2021), these methods require proper sensor placement and additional user training on sensor usage. A more practical alternative lies in camera-based solutions, which capitalize on a common behavioral cue associated with distraction – looking away from the task. Research in other domains (Zaletelj and Košir, 2017; Smith et al., 2003; Prajod et al., 2023) has indicated that facial and body pose features, including gaze direction, head orientation, and body posture, can effectively detect loss of attention. Inferring attention from such features is contingent on the setup (e.g., screen position), and detection models need to be appropriately calibrated. Nonetheless, this approach presents a cost-effective and unobtrusive solution when compared to sensors like EEG.
  Since attention/distraction detection models rely on the setup layout and positioning of objects, a direct comparison of their performance is difficult. However, models trained on public distraction datasets have been reported to achieve high performance. For instance, studies have reported convolution neural networks (CNNs) achieve accuracies higher than 90% on public driver distraction datasets such as State Farm and AUC datasets (Kashevnik et al., 2021).
- **Pain**: Research on the occurrence of pain within the neurorehabilitation population and the consequent necessity for medical interventions has been extensively explored in works dedicated to neurorehabilitation (Benrud-Larson and Wegener, 2000; Castelnuovo et al., 2016). In the realm of healthcare applications, numerous systems employ image or video-based automatic pain detection (Kunz et al., 2017; Sellner et al., 2019). These approaches typically entail the identification of pain based on facial expressions captured by a frontal camera. Some works (Lopez-Martinez and Picard, 2018; Werner et al., 2014) have also delved into the utilization of physiological signals such as ECG, EDA, etc., for pain recognition. Despite recent strides in affective computing toward automatic pain detection, the available datasets remain limited in size, often necessitating techniques like transfer learning to address this constraint (Wang et al., 2018; Prajod et al., 2021).
  The UNBC-McMaster shoulder pain dataset (Lucey et al., 2011) and the BioVid heat pain dataset (Walter et al., 2013) are widely used publicly available datasets for pain detection. The CNN models trained on UNBC-McMaster have achieved state-of-the-art performance of 91% accuracy Ben Aoun (2024), whereas few models have achieved more than 70% accuracy on BioVid dataset (Werner et al., 2016; Prajod et al., 2022; Gkikas and Tsiknakis, 2023). However, models trained on the BioVid dataset have been shown to be more robust to data from other datasets (Othman et al., 2019; Prajod et al., 2022, 2024b).
- **Stress**: Detecting stress becomes crucial, especially with the introduction of gamification elements in the training session, where the patient may experience stress, particularly if the exercise surpasses their current skill level. Extensive research has explored diverse modalities for stress detection, encompassing physiological signals, speech, gestures, and contextual behavioral patterns (Koceska et al., 2021; Larradet et al., 2020; Giannakakis et al., 2019; Heimerl et al., 2023). Physiological signals, including ECG, BVP, EDA, and respiration, have demonstrated high efficacy in stress detection (Gedam and Paul, 2021; Prajod et al., 2024a; Smets et al., 2018). Audio or speech analysis is another prevalent modality for automatic stress recognition (Dillon et al., 2022; Lefter et al., 2015). However, this approach typically involves substantial verbal interaction with the agent, a scenario not anticipated during neurorehabilitation exercises. Contextual behaviors, such as keystrokes and specific gestures, are often tailored to specific use cases and may not be directly applicable in the context of neurorehabilitation.
  The WESAD dataset (Schmidt et al., 2018) is a popular stress datasets which includes high arousal positive state (amusement) and negative state (stress). Both these states may occur during the training session, depending on the difficulty level of the exercise. Previous works have demonstrated high-performing stress detection models (accuracy: 83%–93%) trained on the WESAD dataset (Vos et al., 2023).

### 2.4 Warmth and competence

In the pursuit of developing socially interactive agents for neurorehabilitation, our aim is to not only create effective agents but also ensure that they are perceived as warm and competent. These perceptions of warmth and competence hold substantial importance as they underpin the establishment of trust and user engagement, both of which are integral to the success of our approach. In these terms, several key factors emerge as critical determinants of success. Anthropomorphism, which involves attributing human-like qualities to non-human entities, plays a fundamental role in cultivating a sense of warmth in social agents (Nass and Moon, 2000; Lee et al., 2006). Users tend to respond more positively to agents that exhibit anthropomorphic traits, perceiving them as approachable and friendly, and this perception of warmth significantly contributes to users' overall experiences and their willingness to cooperate with the technology (Prajod et al., 2019).

Competence, another essential factor, has been identified in psychology research as a critical determinant of trust (Hancock et al., 2004; Bickmore et al., 2009). An agent's competence, reflecting its capabilities and effectiveness, directly influences the trust users place in it (Hancock et al., 2004; Bickmore et al., 2009). Users are more inclined to trust and cooperate with agents they perceive as competent in assisting them with their rehabilitation tasks. Both warmth and competence exert substantial influence on user engagement, as research demonstrates that users are more engaged and motivated to interact with agents perceived as both warm and competent (Nass and Moon, 2000). This heightened engagement is of paramount significance in neurorehabilitation, as it bolsters users' commitment to therapy and increases the likelihood of positive outcomes (Nass and Moon, 2000).

To enhance the warmth and competence of social agents in the context of neurorehabilitation, careful consideration must be given to anthropomorphic design elements, including the incorporation of human-like features, gestures, and verbal and non-verbal communication styles, all of which may elicit feelings of warmth and trust in users (Nass and Moon, 2000; Lee et al., 2006). Our research underscores the importance of integrating anthropomorphic design, effective communication strategies, and a robust knowledge base in crafting agents that not only provide effective assistance to patients but also cultivate trust, foster engagement, and contribute to positive rehabilitation outcomes. It is evident that further investigation is warranted to delve deeper into the nuances of how warmth and competence perception influence user engagement and, ultimately, the outcomes of neurorehabilitation interventions.

### 2.5 Socially interactive agents as medical coaches

In the realm of neurorehabilitation, the use of socially interactive agents as medical coaches has garnered increasing attention due to their potential to enhance patient engagement and therapeutic outcomes. This section provides an overview of related works in this domain, shedding light on the role of virtual coaches and their impact on patient motivation and progress.

Within the domain of virtual coaching and interactive systems for medical applications, several notable initiatives have paved the way for the development and implementation of socially interactive agents in various healthcare contexts. Bickmore et al. (2005) introduced the Fit Track system, featuring the relational agent Laura, who serves as an exercise advisor. Laura engages with patients, motivating them to participate in physical activities and thereby fostering their rehabilitation progress. This system stands as an early exemplar of virtual coaching in the medical field. The SenseEmotion project (Velana et al., 2017) explored pain management strategies among the elderly, employing an avatar for crisis interventions to facilitate reassuring dialogues and support for older adults. This initiative highlighted the potential of avatars in the context of pain management. Schneeberger et al. (2021) delved into stress management using virtual characters in simulated job interview scenarios. Their research aimed to understand the various interaction strategies these virtual characters could employ to induce stress in participants, offering valuable insights into human-agent interaction and applications in training and preparatory systems. Neumann et al. (2023) investigated the impact of virtual social support on physiological pain responses within a virtual reality environment, showcasing the potential for virtual characters to provide emotional support and reduce physiological pain responses. Additionally, Giraud et al. (2021) proposed a tangible and virtual interactive system to train children with Autism Spectrum Condition in joint actions, demonstrating the broader potential of socially interactive agents in training social and motor skills relevant to neurorehabilitation. Nadine, a Digital Human Cardiac Coach, was developed to support heart patients throughout their cardiac health journey. Considering the specific application of avatars during neurorehabilitation therapy, a number of examples exist in literature but most of them introduce the virtual character with a first-person strategy, with the aim of giving a more realistic visual feedback to the patient. Examples of this kind of approach can be found in works such as Cho et al. (2017), where the authors introduce a first-person avatar in combination with a Functional Electrical Stimulation (FES) system achieving promising results. Moreover in de Sousa and Balbino (2018), a lower-limb rehabilitation robot assists Spinal Cord Injury (SCI) patients to perform their exercises while a first-person avatar feedback is provided inside a virtual environment. Along the same lines, Gümüslü et al. (2021) exploits a first-person avatar to explore virtual environments with the aim of enhancing patient engagement during neurorehabilitation treatment with a Lokomat rehabilitation system.

In this study, we strategically choose to build upon the Gloria biofeedback training system, a well-established platform developed by our Affective Computing group. By leveraging Gloria's robust framework, we ensure continuity in technological advancement and capitalize on its pre-existing integration capabilities with our current methodologies. This deliberate choice is guided by the system's demonstrated success (Schneeberger et al., 2021), providing a solid foundation for adaptation of its behavior and training strategies to specifically address the unique requirements of neurorehabilitation. Our primary objective is to conceptualize a comprehensive system that seamlessly integrates a motivating virtual coach with a robotic rehabilitation device to optimize patient engagement and enhance rehabilitation outcomes. Through this exploration, we aim to address the unique challenges and opportunities presented by neurorehabilitation, emphasizing the potential of socially interactive agents as valuable allies in the journey toward patient recovery. By synthesizing these related works, we draw inspiration from the successes and insights offered by virtual coaches in various medical domains, applying them to the specialized context of neurorehabilitation and striving to empower patients and facilitate their path to recovery.

## 3 Materials and methods

**Figure 2:**
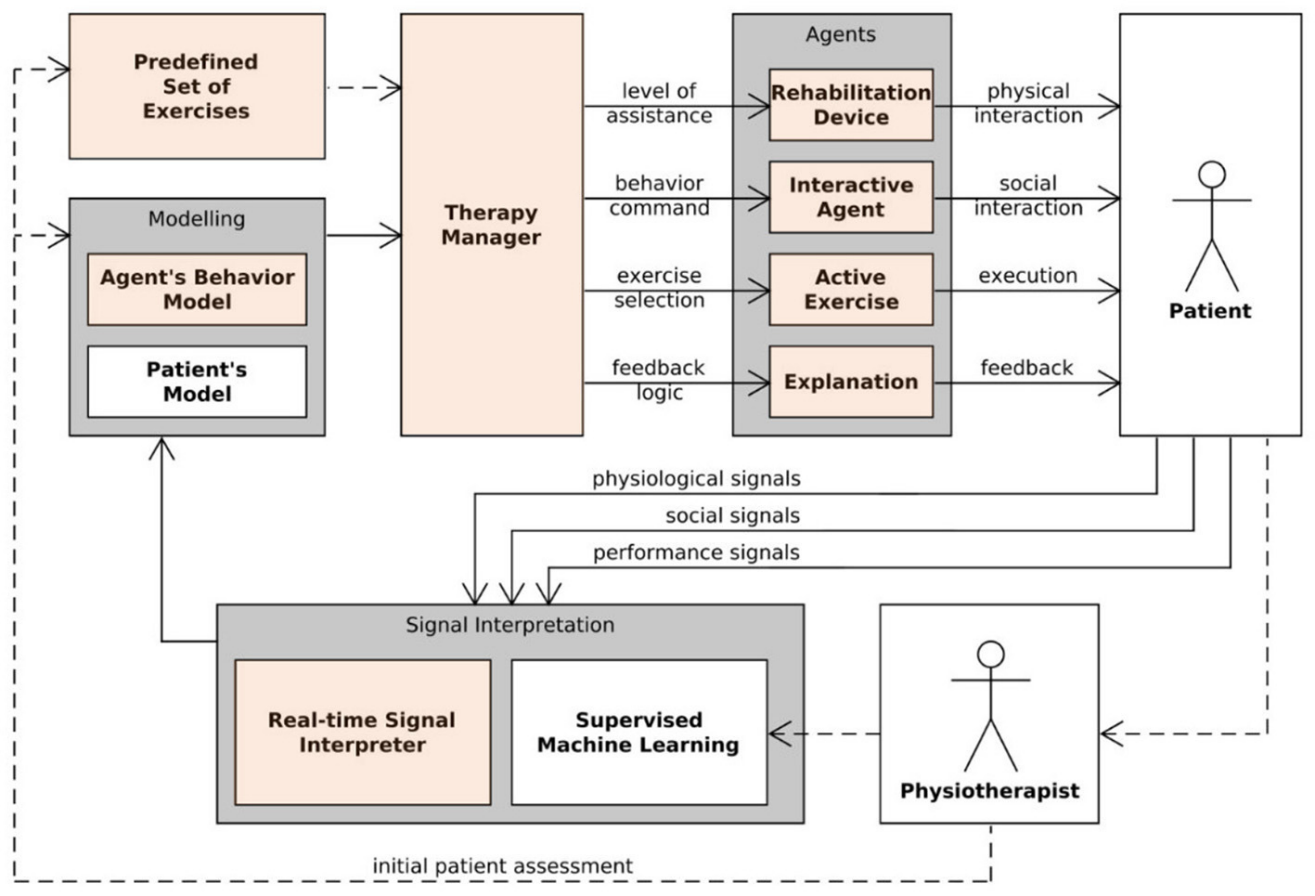
Human-in-the-loop Empathetic Neurorehabilitation Trainer concept.

The envisioned Empathetic Neurorehabilitation Trainer system is shown in Figure 1, built on the basis of the architecture depicted in Figure 2. The system is equipped with a robotic *Rehabilitation Device* and a virtual socially *Interactive Agent*. Both device and agent can adapt their behavior based on the *Patient*'s performance, as in most assistance-as-needed paradigms, and it also takes into account the patient's affective state. Moreover, these two entities, together with the specific task to be carried out and the feedback media chosen to provide the patient with an *Explanation* regarding the *Active Exercise*, are intended to work as a single entity, actively collaborating to improve the rehabilitation session outcomes further.

### 3.1 Concept

With reference to Figure 2, all software and hardware components make up a closed-loop architecture where the monitoring of a set of heterogeneous parameters is introduced. In fact, during the execution of the task, the robotic device is in charge of collecting data regarding the kinematics of the patient's movement (e.g., position, speed), and a wearable device (e.g., Polar Band) is used to extract various physiological signals (e.g., ECG, EDA). At the same time, a camera captures the patient's upper body for behavioral signal interpretation purposes. These raw data represent the input for a *Signal Interpretation* module, responsible for providing a series of higher-level quantities such as patient's performance, attentiveness, and stress. As depicted, the *Physiotherapist* still has a central role in the proposed approach. In fact, professional expertise is required for the patient's initial assessment (e.g., residual mobility, attention span), used to define the backbone of both a *Patient's Model* and an *Agent's Behavior Model* and to prepare a *Predefined Set of Exercises*. Moreover, a *Supervised Machine Learning* module is employed to learn from the physiotherapist how to optimally balance the target execution performance for the exercise and the social experience for the specific patient. Also, both challenging and entertaining portions of the session must be included to maximize the patient's attention. Closing the loop, a *Therapy Manager* actively exploits the inferred information to decide how the behavior of the socially interactive agent should be changed, which explanations should be given, and which exercise and difficulty level should be activated to optimize the therapy experience and effectiveness.

Considering Figure 2, the highlighted modules are the ones that were not only conceptualized but completely realized and tested with a small set of volunteers to verify the correct functioning of all the components. A detailed description of those modules is provided in the following sections.

### 3.2 Robot control

**Figure 3:**
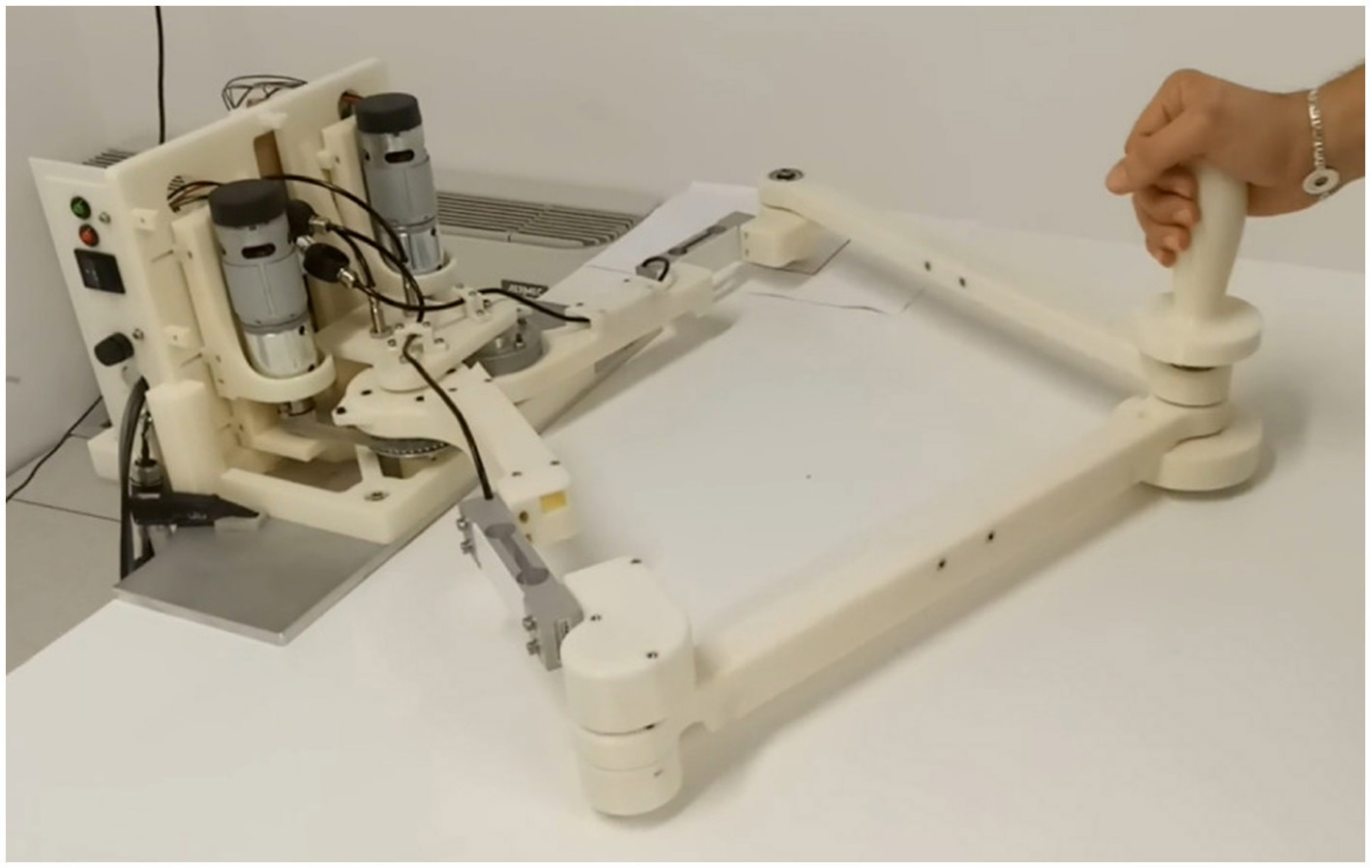
The PlanArm2 prototype.

As already mentioned, the backbone of the presented architecture is based on the Robot Operating System (ROS) (Quigley et al., 2009). In particular, the whole robot control software has been realized using ROS Noetic on an Ubuntu 20.04 machine, leveraging the built-in functionalities of *ros_control* (Chitta et al., 2017) and *MoveIt!* (Coleman et al., 2014). Thanks to this approach, the whole software architecture developed in this study is independent of the choice of specific robotic device selected for the rehabilitation practice. However, in order to validate its correct functioning, the system is tested on a prototypical device called PlanArm2 (Yamine et al., 2020). This 2-DOF planar upper-limb rehabilitation robot, depicted in Figure 3 is selected because of its affordable and compact design, perfectly suited for home-based therapy applications. In simplified terms, the implemented robot control system waits for a command containing the trajectory to be executed by the patient as part of the exercise. The latter is defined by the therapist using a dedicated Graphical User Interface (GUI), presented in Section 3.3, and sent to the active controller. As the patient starts moving the robot handle along the predefined trajectory, the controller monitors the current handle position with the relative ideal position on the trajectory and generates an assistive restoring force if this error overcomes a certain threshold. During the exercise, the actual position of the robot handle is also communicated back to the GUI both for generating a visual feedback for the patient and for monitoring the execution performance.

### 3.3 Graphical user interface

**Figure 4:**
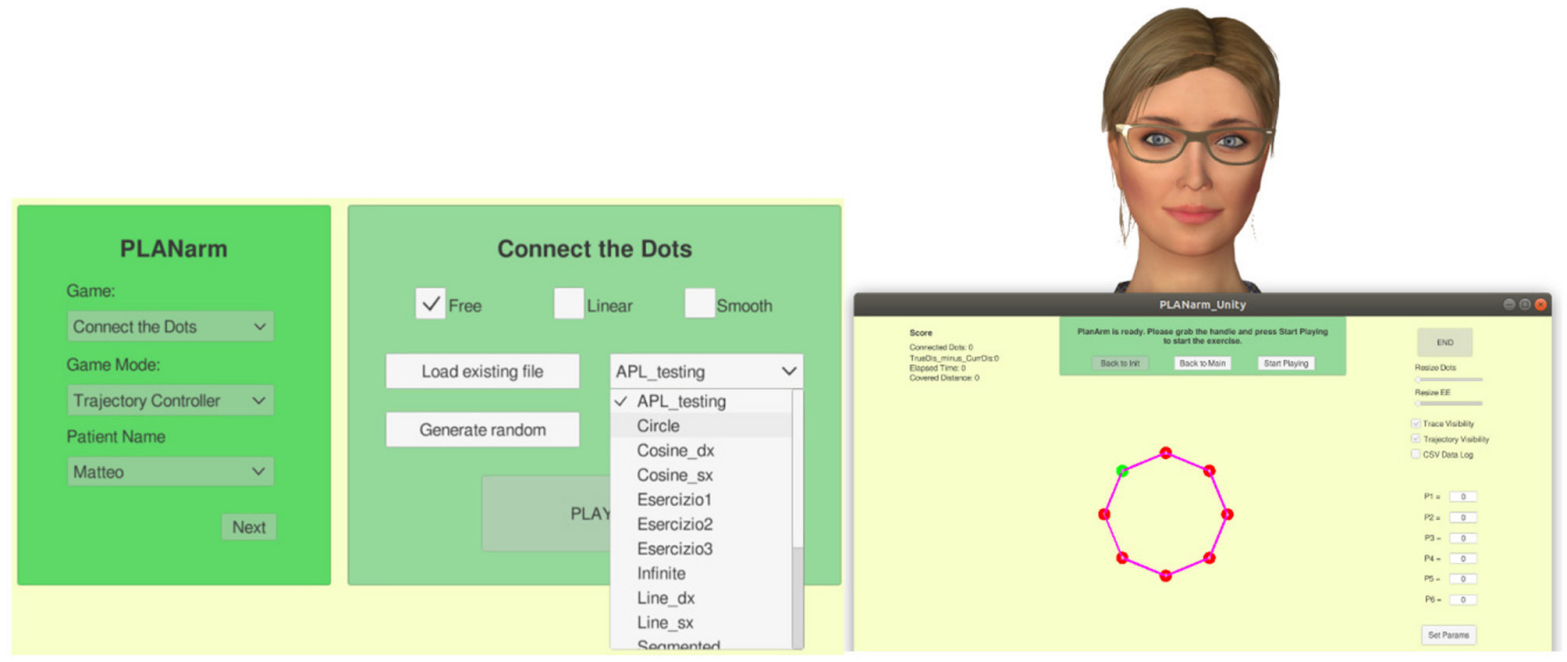
On the left a screenshot of the game and assistance selection page. On the right a screenshot of the visual feedback provided to the patient together with the virtual character.

In this study, participants engaged in a series of training exercises designed to improve their precision and control in a virtual environment. The training was facilitated through a graphical user interface (GUI) Figure 4 developed using Unity3D (Haas, 2014), which was connected to a robotic system via ROS (Robot Operating System) (Younesy, 2022). The exercises were presented as interactive games, each guided by a virtual coach named Lydia. The training consisted of three distinct games: Circle, Infinity, and Line (Figure 3). In each game, participants were required to follow a predefined path (a circle, an infinity symbol, or a straight line) on a plane. These paths were marked by a series of dots, and participants used the PlanArm2 device (Section 3.2) to apply the appropriate amount of force to reach each dot as accurately as possible. As the patient starts moving the robot handle along the predefined trajectory, the controller continuously monitors the current handle position relative to the ideal position on the trajectory. If the deviation exceeds a certain threshold, the controller generates an assistive restoring force to guide the patient back on track. Additionally, the actual position of the robot handle is communicated back to the GUI in real-time, providing visual feedback to the patient and allowing for precise monitoring of execution performance. This setup simulates a real-world physical therapy scenario, where precise control and adherence to a prescribed movement trajectory are critical.

A small pilot study was conducted with 18 healthy participants, including 12 men and 6 women. The sample comprised 7 undergraduates and 11 master's students, with ages ranging from 22 to 33 years (M = 26.11, SD = 2.87, median = 25). All the participants were healthy adults and did not suffer form any neurological impairments. There were no specific inclusion or exclusion criteria as the study aimed to assess the system's baseline usability and engagement.

Each participant completed three sessions of the selected exercise, with each session lasting approximately 15 minutes. During these sessions, several key performance metrics were recorded in real-time: Trajectory Error (the deviation from the ideal path), Distance Traveled, Time Taken to complete the task, and Tracking Error (which informs the user if they moved away from the given ideal trajectory). These metrics were meticulously recorded and transmitted to the visual scene maker (VSM) for analysis (Gebhard et al., 2012). In addition to tracking the physical performance, Lydia also monitored the participants' social signals, including signs of stress, pain, or loss of attention (Section 3.4). If any social signal exceeding the normal threshold was detected during a session, such as stress, pain, or attention lapses, the system automatically intervened after the session, communicating the detected signal to the participant and offering suggestions to overcome it, such as taking a pause. Lydia adapted its behavior based on the detected signals, ensuring personalized feedback. After each session, Lydia provided participants with a summary of their performance and any detected social signals. At the end of the training, Lydia also communicated the session in which the participant demonstrated the best precision, offering a comparative analysis of the three sessions. The primary goal of these training games was to enhance the participants' motor control and precision through repetitive, targeted exercises, while also providing real-time feedback to both the participants and the therapist. The interactive nature of the games, combined with the feedback from Lydia, was designed to engage the participants and ensure that they remained focused and motivated throughout the training.

### 3.4 Machine learning models

**Figure 5:**
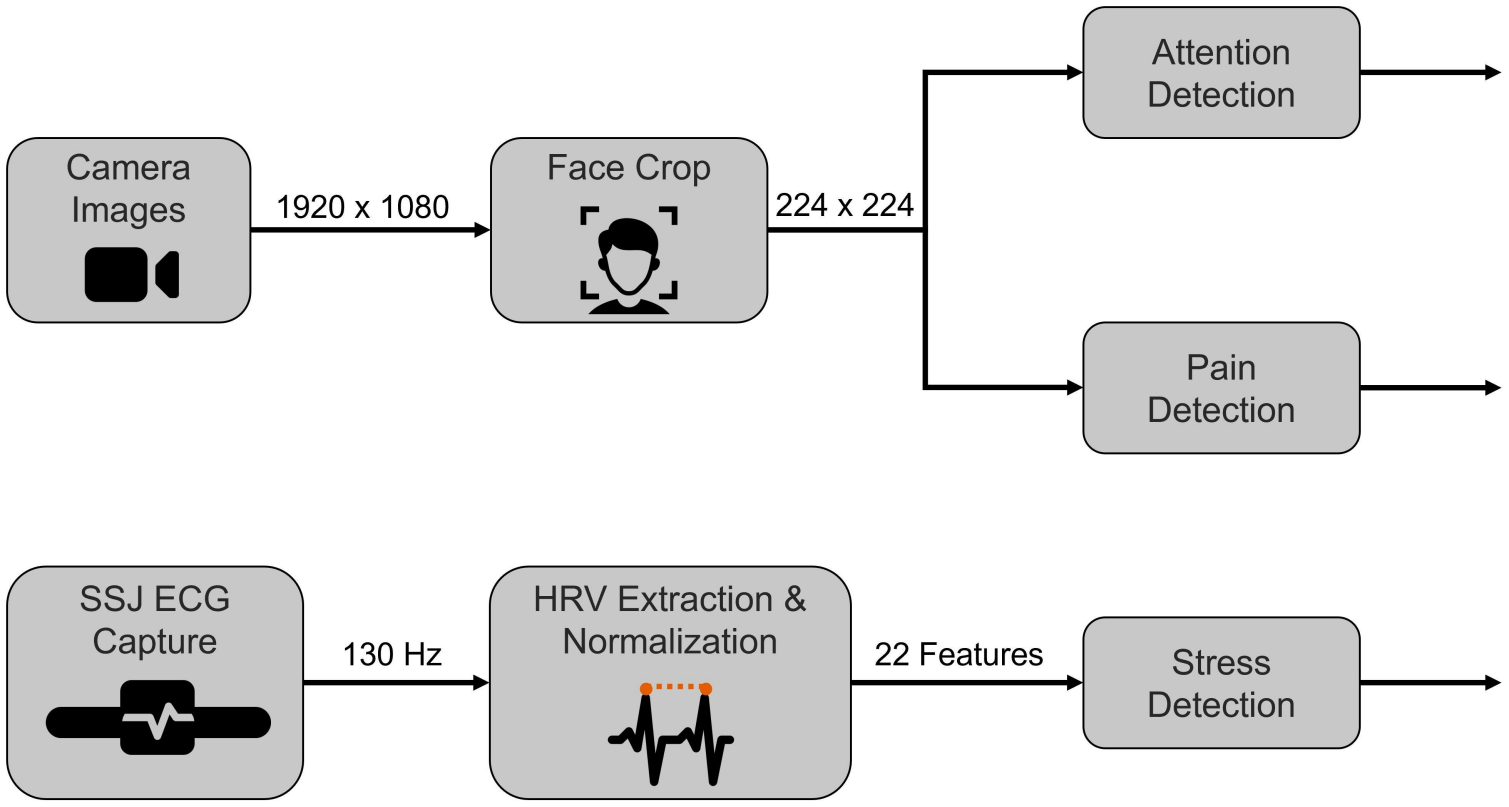
Illustration of pipelines deployed for real-time prediction of attention, pain, and stress. This image was created using resources from Flaticon.com.

Various affective and physiological signals can be used to analyze the mental and physical states of the user. We trained machine learning models to detect distraction, pain, and stress. To facilitate the real-time prediction of the user states, we employ two existing frameworks—SSI (Wagner et al., 2013) and SSJ (Damian et al., 2018). SSI is a Windows-based framework, whereas SSJ is developed for Android. The pipelines involving facial images including capture, processing, and predictions (attention, pain detection) are implemented using SSI. We use the SSJ plugins to capture the raw ECG signal from the Polar H10 device and stream it to SSI. SSI receives the raw ECG signals from SSJ and the subsequent pre-processing, feature extraction, and stress detection steps are performed within the SSI pipeline. Figure 5 visualizes the SSI pipelines that were implemented and deployed. The per-frame classification outputs from the models are communicated to the agent via UDP (User Datagram Protocol) sockets.

#### 3.4.1 Attention detection

The patient may become distracted during the training session due to boredom or lack of motivation. In neurorehabilitation, the patient's level of attention is a crucial input for the agent's motivational strategy. Distraction or lack of attention is a key state to detect in this context. We say a user is distracted when they are paying attention to the surroundings rather than the training exercise screen. We can redefine the problem as a use case of gaze estimation, where the user's gaze on the screen is considered attentive and anywhere else is considered distracted.

We train a deep neural network for discerning attention to screen and distraction following the approach proposed by Prajod et al. (2023). First, we train a VGG16 network for gaze estimation using the ETH-XGaze dataset (Zhang et al., 2020). This dataset has high variations in gaze, including extreme head positions. The input images are face-cropped by leveraging the face detection model (Bazarevsky et al., 2019) provided by MediaPipe. The input images are scaled to the default VGG16 dimensions of 224 × 224. The network outputs pitch and yaw values corresponding to the gaze direction.

Next, we need a target dataset for training the network on our specific task. We collected a small dataset (approximately 200 images) of people looking at the screen and distracted (looking away in random directions). Five participants (3 males, and 2 females; aged 18–30 years) took part in the data collection, of which three wore glasses. High-resolution images (1,920 × 1,080) were captured using a camera positioned on top of the screen.

Finally, we adopt a transfer learning approach to detect when the user is distracted. We fine-tune the prediction layer of the gaze estimation network using the collected dataset. The prediction layer is adjusted for 2-class classification (screen or away) and uses Softmax activation. Similar to the gaze estimation network, the input images are face-cropped and scaled to 224 × 224. The fine-tuning is performed using SGD optimizer (learning rate = 0.01) and categorical cross-entropy loss function. We follow leave-one-subject-out (LOSO) evaluation to train the model using data from four participants and reserve the unseen data from one participant for validation. Our model achieves an average accuracy of 84.6%.

In the real-time setup, we use a frontal face camera (Logitech RGB camera) to capture the facial expressions of the users. Each image from the video sequence is passed through a face crop plugin which crops the image to the face region and scales it to 224 × 224. The pre-processed image serves as input to the attention and pain detection (detailed below) models.

#### 3.4.2 Pain detection

Pain assessment and management are crucial for medical interventions in neurorehabilitation and hence, is a state that needs to be monitored during the therapy session. In this work, we train a deep-learning model that can discern pain and no-pain images from facial expressions captured by the front camera. One major challenge here is that pain datasets are typically small for training deep learning models (Wang et al., 2018; Hassan et al., 2019; Xiang et al., 2022). To circumvent this, we follow the transfer learning approach described by Prajod et al. (2021), which involves leveraging features learned for emotion recognition in pain detection. To this end, we train an emotion recognition model using a large dataset called AffectNet dataset (Mollahosseini et al., 2017). The model uses a VGG16 network to classify an input image as Neutral, Happy, Sad, Surprise, Fear, Anger, Disgust, and Contempt.

To adapt this model for pain detection, we fine-tune the model using images from a pain dataset. We consider two datasets commonly used in automatic pain recognition - UNBC-McMaster shoulder pain expression database (Lucey et al., 2011) and BioVid heat pain dataset (Walter et al., 2013). Models trained on these datasets have been shown to learn well-known facial expression patterns of pain (Prajod et al., 2022). So, we use a combined dataset by merging UNBC and BioVid datasets. This increases the samples available for training a pain model. However, both these datasets are derived from video sequences and thus, have virtually repetitive images. To mitigate this redundancy, we select the images following the strategy proposed by Prajod et al. (2022). We also leverage the training, validation, and test dataset split that they proposed.

The prediction layer of the emotion recognition model is modified for a 2-class prediction of pain and no-pain classes. The entire network is fine-tuned using the combined pain dataset. Like in the case of attention detection, the images are face-cropped and scaled to the default VGG16 dimensions. The fine-tuning process employed an SGD optimizer (learning rate = 0.01) and focal loss function. The model achieved an average accuracy of 78% on the test set.

#### 3.4.3 Stress detection

The patient may experience stress while performing the exercise, especially if they find the exercise hard or if they are unable to complete the recommended exercise. Such negative experiences can severely impact the user's level of motivation and their willingness to continue training. Hence, the user's stress level is an important input to the agent.

Unlike the other models, we rely on hand-crafted HRV (Heart Rate Variability) features to detect stress. This choice is based on the observations presented by Prajod and André (2022), where HRV features showed more generalizability than models based on raw ECG signals. We used the ECG signals from the WESAD dataset (Schmidt et al., 2018) to derive the HRV features for training the stress detection model. This dataset contains physiological signals collected from 15 participants during a social stress scenario. We compute 22 HRV features from the time domain, frequency domain, and poincar plots. These features are computed using 60-second-long ECG segments. The pre-processing steps and feature extraction are detailed in Prajod and André (2022).

We trained an SVM (Support Vector Machine) with the radial basis kernel function to predict if the user is stressed or not. To mitigate the individual differences in the signal and derived features (e.g., resting heart rate), the signals undergo MinMax normalization. This model achieves an average accuracy of 87% in LOSO evaluation.

The trained model is incorporated into the real-time pipeline (see Figure 5). We use the Polar H10 chest band to collect ECG signals. The data from the initial 5 minutes is considered as baseline data and the corresponding HRV features are used to compute the normalization parameters for each user. For the subsequent data, we compute the HRV features, normalize them, and detect stress using our SVM model.

### 3.5 Interactive agent

**Figure 6:**
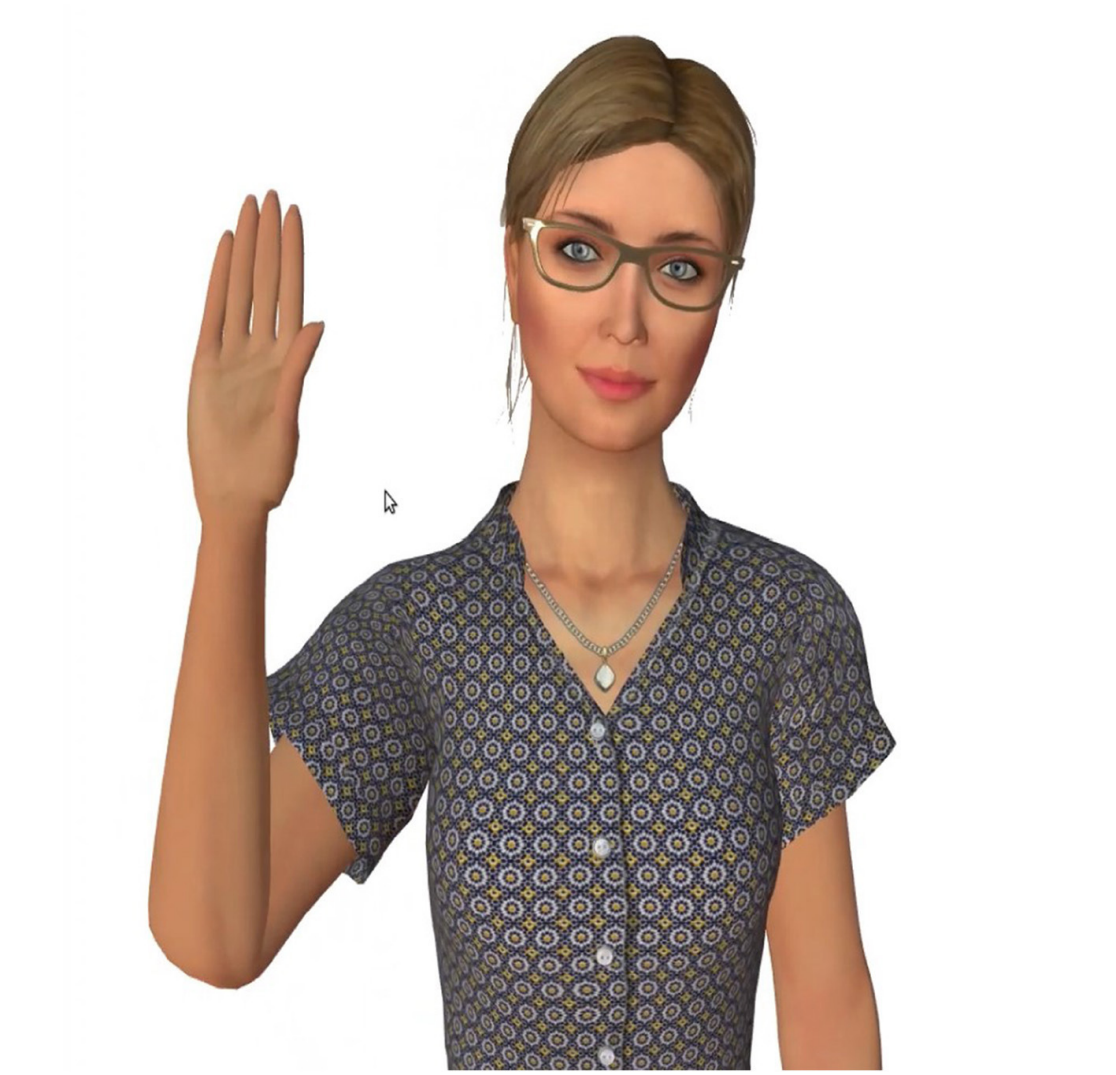
Socially interactive agent, Lydia.

A motivating agent is used in this paper to support the patients during their training sessions (Figure 6). The agent is displayed on a monitor along with the training exercises and instructions (refer to Section 3.3). The agent serves as a coach, motivating, informing, and assisting the patient with certain neurorehabilitation tasks. As stated in the introduction, it would be important that the agent's behavior and the rehabilitation device's behavior are calibrated in such a way that they appear to be one entity, with the agent assisting the patient in applying a particular amount of force through the physical capabilities made available by the robot.

The decision to use Lydia was further supported by research. For instance, Schneeberger et al. (2021) conducted a study on stress management training using biofeedback guided by social agents, where a similar agent, Gloria, was effectively employed to help individuals manage stress through interactive training sessions. Additionally, research by Gebhard et al. (2014) explored interaction strategies for virtual characters to induce stress in simulated job interviews, further demonstrating the effectiveness of socially interactive agents in training scenarios. This body of work demonstrates that such agents are effective in guiding users through training programs, particularly by providing a dynamic and responsive interaction experience that enhances learning and engagement.

Lydia is a socially interactive agent equipped with advanced speech synthesis and animation capabilities, allowing her to engage in lifelike, contextually appropriate interactions based on social cues. Her speech output is generated using the Nuance Text-To-Speech system, which supports precise lip-syncing and manipulation of speech patterns. To enhance realism, Lydia's animations are controlled through direct manipulation of her skeleton model joints, such as the neck and spine, enabling nuanced and dynamic physical responses (Gebhard et al., 2014). She can perform 54 conversational gestures captured via motion capture technology, adjustable in real-time, and express a range of 14 facial expressions, including the six basic emotions defined by (Ekman, 1992). Lydia's speech patterns and gestures are dynamically aligned with the user's recognized social signals, as identified by our system's social signal interpretations, with the Scenario Manager selecting the appropriate reactive behavior model to ensure contextually suitable and engaging interactions.

Additionally, Lydia's design incorporates human-like features and behavior, including narrow gestures, positive facial expressions, shorter pauses, and friendly head and gaze behavior. On the verbal level, explanations and questions show appreciation for the user and contain many politeness phrases (Gebhard et al., 2014). The interactive agent is designed to follow the best practices of training professionals (Section 2.1). Our approach includes the deliberate incorporation of human-like features and behavior in the virtual agent's design, thereby establishing an immediate connection with users. This anthropomorphic design choice goes beyond aesthetics; it serves as a conduit for users to attribute human-like motivations and intentions to the agent, reinforcing feelings of warmth and approachability. This parallels the significance of warmth and competence in human-human relationships and leverages the concept that individuals often apply the same social rules and expectations to virtual agents as they do to humans (Nass and Moon, 2000; Epley et al., 2007).

At the heart of our agent's design and implementation lies the fundamental concept of trust. Drawing inspiration from established principles of trust in human-human relationships, we have meticulously integrated key elements of warmth and competence into our virtual agent's behavior. To ensure that our agent embodies trustworthiness, we partnered with experienced psychologists and employed advanced tools like the VSM. Through iterative refinements, we fine-tuned the agent's verbal expressions to strike the right balance between warmth and competence. These interactions serve as a tangible representation for making the agent a trustworthy ally in the therapeutic journey.

**Figure 7:**
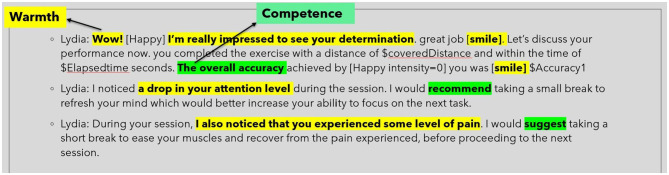
Words and phrases that portrays the agent as warm and competent.

Our agent effectively balances warmth and competence in communication. Warmth is conveyed through expressions like “WOW!” and commendations such as “I'm impressed by your determination” (see Figure 7). In contrast, its competence is highlighted with terms like “overall accuracy,” and action verbs like “suggest” and “recommend” (Gebhard et al., 2014). The agent employs gestures, nods, and verbal affirmations, including smiles, to bolster user engagement and cultivate a conducive training atmosphere.

**Figure 8:**
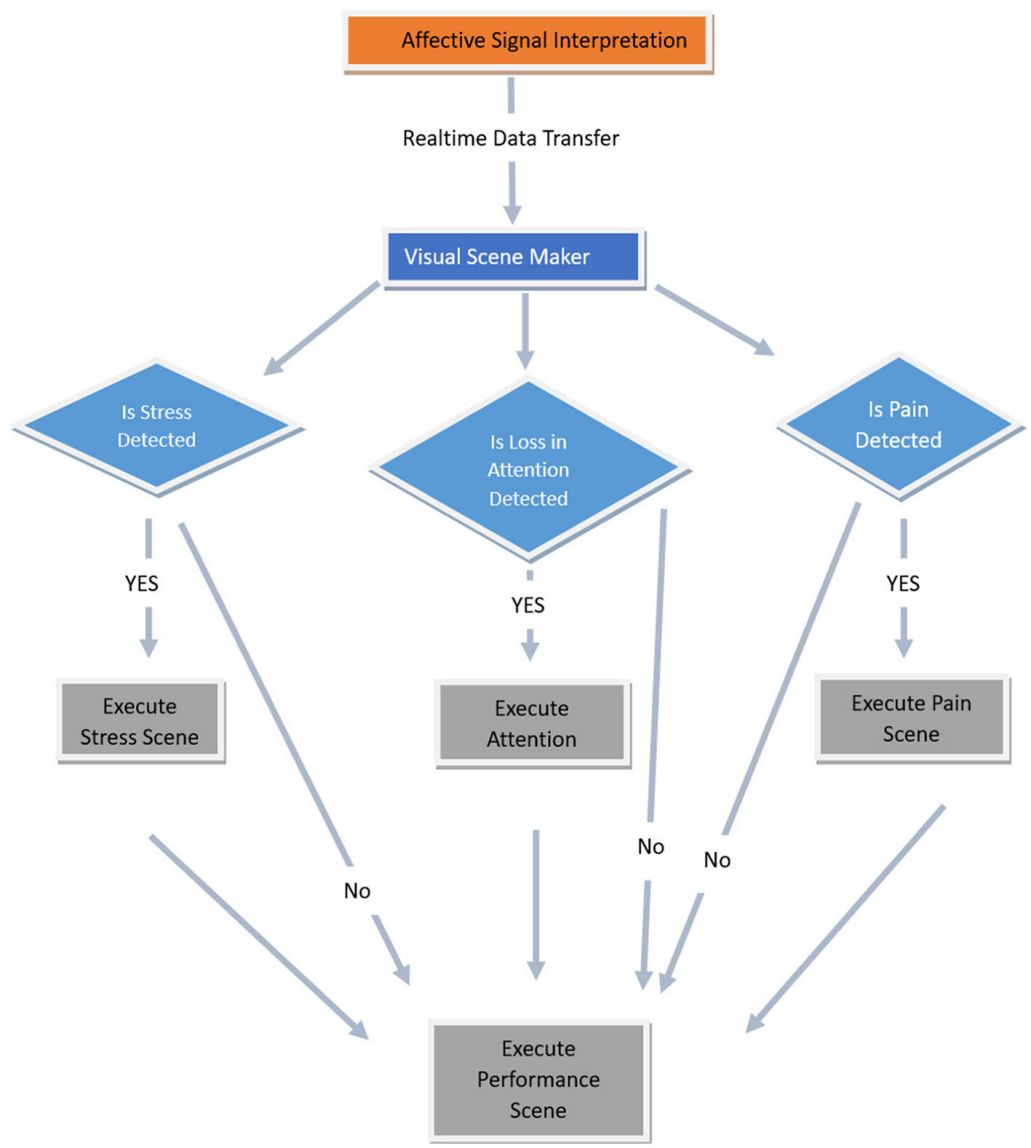
Flow chart showing how the affective signals are utilized in executing different scenarios in VSM.

The agent's dynamism lies in its adaptability, seamlessly tailoring its behavior according to the user's physiological and affective cues. These cues, processed in real-time by a signal interpretation framework, enhance the agent's therapeutic relevance (Charamel, 2023). In the context of neurorehabilitation, metrics such as attention and pain are crucial. Hence, an empathic agent capable of identifying attention and pain contributes to the establishment of a rehabilitation environment that minimizes stress, proving essential for sustaining patient motivation. The interactive agent receives affective cues regarding stress, attention, and pain directly from the SSI pipeline into the VSM (Figure 8). If the value of any of these social cues exceeds its threshold, the agent is programmed to empathetically inform the user about their current state.

Hardware-wise, the interactive agent runs on a PC running MS Windows 10TM and operates autonomously in a web browser, interacting via social cues (Charamel, 2023), showing the agent at a realistic size. It uses the CereProc Text-To-Speech system for voice outputs (CereProc, 2023). Lydia, the agent, can execute 36 diverse conversational gestures (Schneeberger et al., 2021). Additionally, 14 facial expressions, including Ekman's basic emotions, further its expressiveness (Ekman, 1992). All agent behaviors are streamlined through the VSM toolkit, ensuring dynamic and user-relevant content.

The agent engages with the system through a graphical user interface. Additionally, it proactively monitors users' affective signals (attention, pain, and stress) during the session. If, during the session, any of these social signals are detected, the agent empathically informs the user and suggests measures to mitigate the issue in the future (e.g., recommends taking a break). Following each session, Lydia delivers a comprehensive performance deviation index summary, subtly encouraging users to improve their future engagement (Figure 8).

#### 3.5.1 Ethical approval

The study has been conducted according to the guidelines of the Declaration of Helsinki and approved by Commissione per l'Etica e l'Integritá nella Ricerca of the National Research Council of Italy (protocol n. 0085720/2022 of 23/11/2022). All 18 participants were briefed about the study and the details of data treatment before signing an informed consent from which they can withdraw at any point.

## 4 Results

The primary aim of the study was to evaluate our proposed framework. Specifically, we examined the effect of an interactive agent during therapy sessions on user engagement and gathered preliminary feedback on interaction quality for potential refinement.

**Figure 9:**
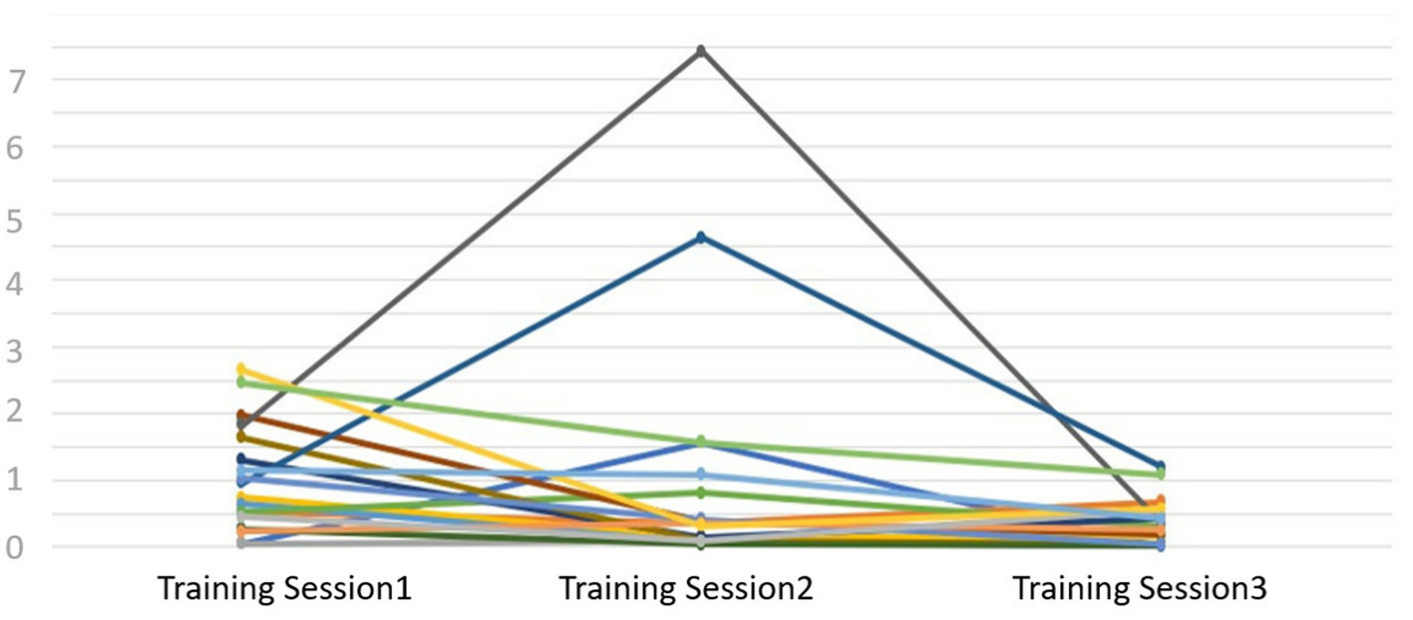
Flow chart showing how the affective signals are utilized in executing different scenarios in VSM.

Figure 9 illustrates the performance deviation index of all participants in the training game. The y-axis represents the performance deviation index scores recorded during the training sessions (refer Section 3.3). The term “performance deviation index” is assessed based on three main criteria: (1) It is defined as the deviation of the actual path followed by the participant from the ideal path and is inversely proportional to the user's performance (2) the total distance traveled, and (3) the elapsed time. Figure 9 specifically utilizes the performance deviation index to compare the performance of all participants. Excluding two notable outliers from Figure 9, there's a consistent downward trend in participants' overall performance deviation index throughout the study's duration. This suggests participants effectively adapted to the device, and the inclusion of the avatar did not negatively impact their performance. In other words, the avatar did not serve as a distraction to the participants during the training sessions. Additionally, the lower the performance deviation index, the better the performance. Therefore, in Figure, we see that the majority of the participants perform better in the third session, which means the performance deviation index is the lowest (i.e., close to zero) for the third session.

In addition to these results of the training game, participants were administered a post-training questionnaire, yielding further insights into their experiences. The results from our pilot study, conducted with 18 healthy participants, provide compelling statistical evidence that the presence of the socially interactive agent, Lydia, significantly enhanced user engagement without acting as a distraction. The study involved quantitative assessments of participants' engagement in the training session.

**Figure 10:**
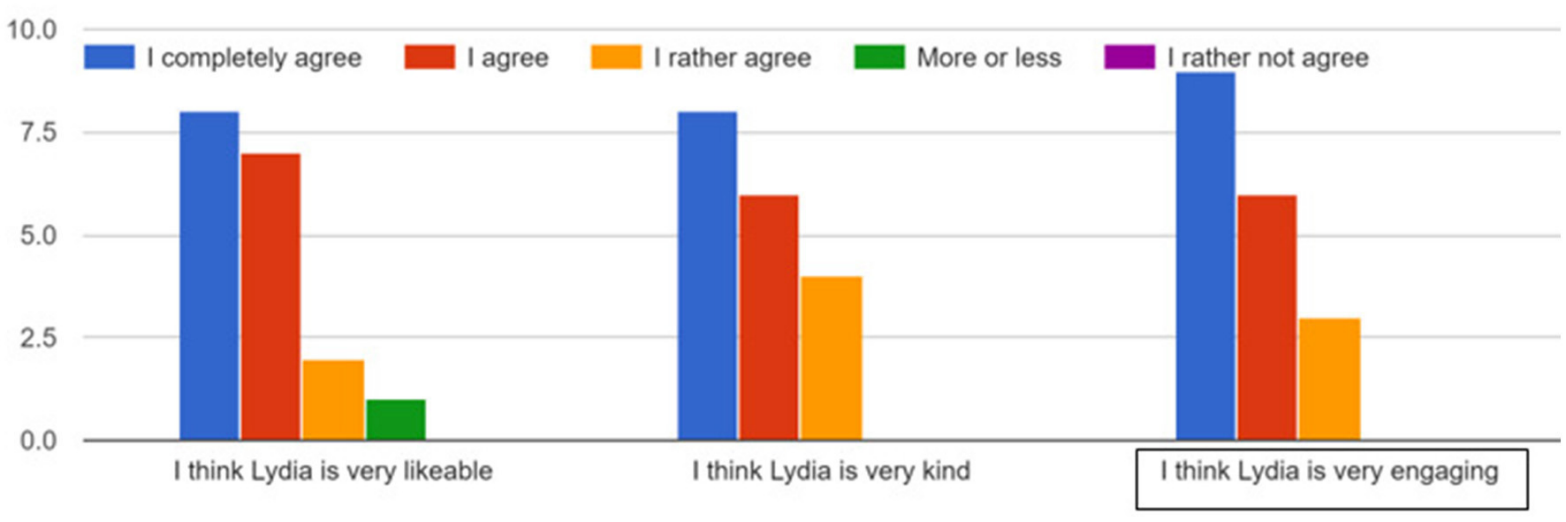
Participant agreement with statements regarding the socially interactive agent Lydia.

**Figure 11:**
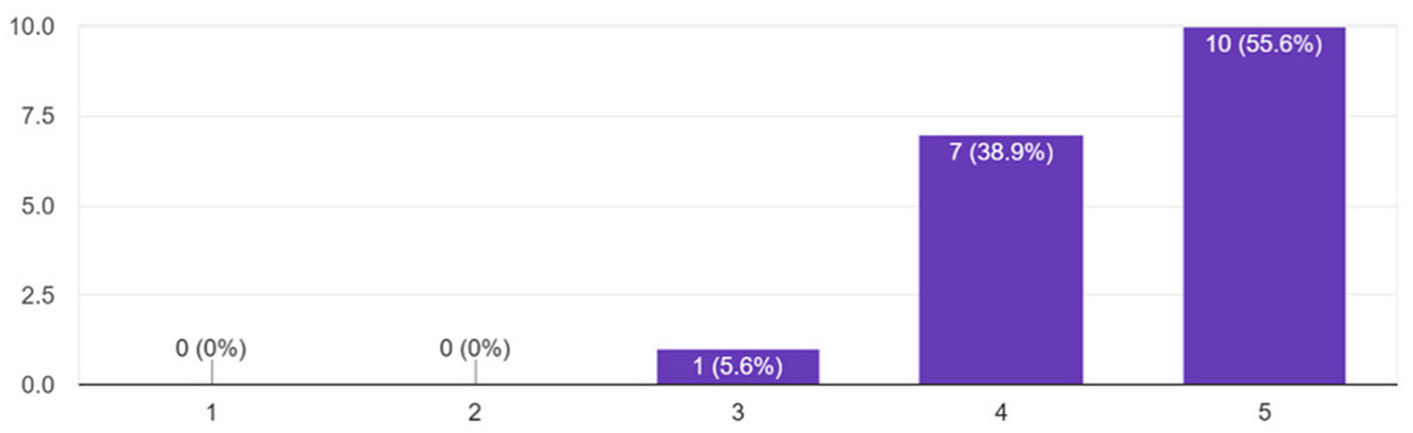
Participants' ratings of the agent's effect on overall engagement in training sessions.

**Figure 12:**
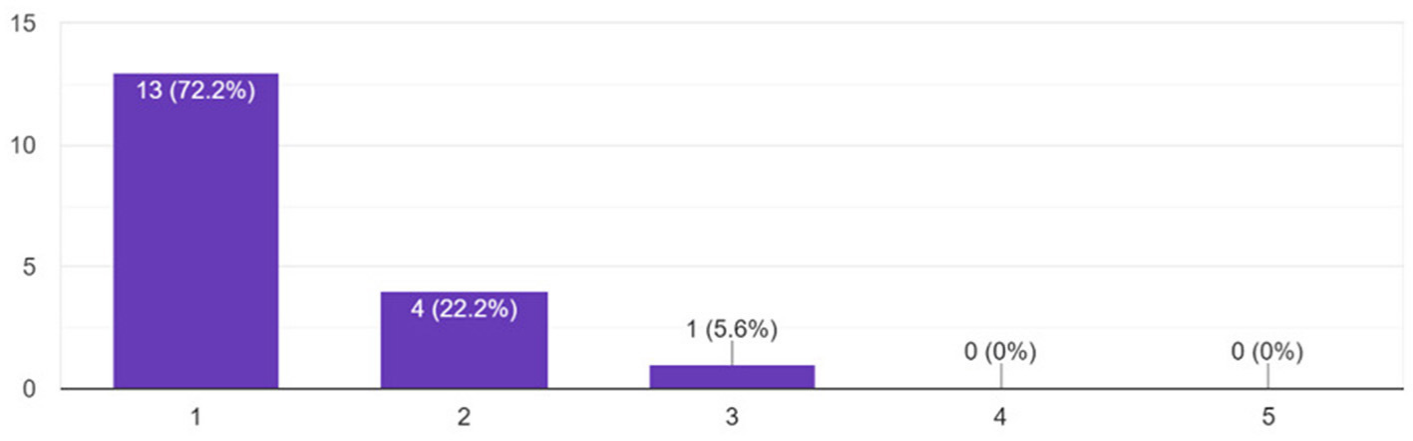
Participants' perception of the agent's impact on performance.

The findings indicate that all participants (100%) found Lydia to be engaging, with 50% of respondents completely agreeing with the statement, 33.33% agreeing, and 16.67% rather agreeing (Figure 10). Overall engagement was also rated highly, with 94.4% of participants assigning ratings of 4 or 5 to the agent's effect on their overall engagement during the sessions (Figure 11). Crucially, the study also assessed whether the agent had any negative impact on participants' performance. The results revealed that 94.4% of participants reported no negative impact, with 72.2% selecting the lowest possible rating (1 on the scale) for negative impact (Figure 12). This result strongly supports the conclusion that Lydia did not distract from the participants' performance but instead contributed positively to their experience.

The pilot experiment also allowed us to identify a series of technical difficulties and limitations that are listed here to ease the reproducibility of the study:

- The Polar chest band needs to be in the right position and in direct contact with the participant's skin. For this reason, some time is spent in wearing the device and making sure data is collected correctly which increases the time to be allocated for each session to preliminary activities.
- The face recognition algorithm only produces a valid output when the participant's face is fully visible within the frame. In our experience, this can become a problem in two situations: either the participant moves around enough to cause the face to be out of frame or the participant's hair is styled in such a way that partially covers the face (e.g., loose long hair). To mitigate this it is necessary to make sure that the camera angle is wide enough to allow the participant for a certain degree of movement and to restyle the hair so that it does not cover the face, again adding some time to the preliminary activities.

## 5 Discussion

The observed steady decline in participants' performance deviation index during the therapy sessions, as shown in Figure 9, underscores the potential effectiveness of our framework. This is particularly noteworthy given the novelty of introducing an interactive agent in such contexts. The data indicates a swift adaptation of participants to the device, and importantly, the presence of the interactive avatar did not act as a distraction, which counters some concerns previously raised in literature about interactive agents in therapeutic settings. However, it's imperative to approach these findings with a degree of caution, acknowledging the inherent limitations such as the sample size and study duration. These factors necessitate further extensive research to comprehensively understand the long-term impact and efficacy of such systems in rehabilitation outcomes.

Building upon the current preliminary results, we can say that the work carried out in works such as Cho et al. (2017); de Sousa and Balbino (2018); Gümüslü et al. (2021), is promising in providing clearer and more realistic visual feedback to the patients, therefore enhancing their engagement and their ability to auto-correct themselves. However, this approach does not address the foreseen issue of a lack of social interaction with the therapist in a future scenario where the therapy is performed remotely. On this topic, promising results are presented in Schneeberger et al. (2021), which provided inspiration for the development of the presented concept and feasibility study, but only explored the approach in a stress management application regarding job interviews.

Future research could be done to explore ways to test and refine the system in clinical settings. One proposed direction involves conducting a comparison study with two groups: one using the interactive agent and one without, to isolate the agent's impact on therapy outcomes. Additionally, we plan to test the system with two types of interactive agents' one supportive and the other demanding. The demanding agent would be introduced when patients are provided with activities of higher complexity, allowing us to assess patient reactions to varying motivational approaches.

Moreover, future studies should involve actual patients in clinical settings, which would provide deeper insights into the system's efficiency and applicability in real-world rehabilitation. Integrating natural language processing would enable patients to communicate with the agent through speech rather than relying on typing or clicking, thereby enhancing user interaction. Another crucial aspect is the ability to configure the agent's behavior in advance based on patient preferences, allowing for a more personalized therapeutic experience.

To further refine the system, we aim to incorporate automatic adaptation of exercise difficulty based on patient performance, ensuring that the therapeutic challenges remain appropriately tailored. Additionally, generating detailed reports for medical supervisors would enhance the system's utility in clinical practice by providing healthcare professionals with comprehensive insights into patient progress.

Another aspect from Figure 2 that require further research is generating explanations. As mentioned before, the agent behavior is aimed to establish trust. Incorporating explanations for agent's decisions can improve trust in the system and increase acceptance of the technology. Visual (e.g., highlighting deviations from ideal path) and audio (e.g., agent explaining predictions through its speech), and textual (e.g., displaying summary) explanations are avenues to be explored in future research.

Our pilot study serves as a foundational step for more in-depth exploration in this domain. The encouraging results from this preliminary assessment underscore the importance of refining the interactive agent's features to enhance user engagement and exploring its applicability in various therapeutic contexts. As we continue to bridge the gap between technology and human-centric care, the integration of feedback from healthcare professionals will be pivotal in enhancing the system's efficacy and ensuring its alignment with clinical practices.

In addition to our preliminary findings, insights from interviews with three neurorehabilitation experts provided both validation of our system's potential and a roadmap for further development and refinement. The experts emphasized that for the system to be effective in a domestic rehabilitation setting, it must be compact, easy to install, and adaptable to various home environments, as well as intuitive enough for use by both patients and caregivers especially those dealing with cognitive disabilities. They also highlighted the importance of embedding exercises within a narrative driven, gamified context, allowing the avatar to support the patient by playing a meaningful role in the rehabilitation scenario. This narrative approach, experts agreed, could significantly enhance patient engagement, especially among kids, by fostering a sense of connection and continuity in the therapeutic process. However, the experts cautioned that compliance with prescribed therapy can often be inconsistent at home, particularly for kids. This limitation emphasizes the need for therapist involvement to monitor the correct execution of exercises and track patient progress, as fully autonomous therapy might risk non-compliance or improper exercise execution.

The experts found the proposed system highly relevant within the field of neurorehabilitation, noting that, if implemented robustly, it could fill a gap in the market. Still, they stressed the need for careful tuning of the avatar's behavior to match each patient's preferences, possibly considering the patient's familiarity with video games. In some cases, a supportive, guiding agent is preferable, while in others, a more challenging approach could motivate patients to push their limits. The ability to switch between these behavioral modes is essential for tailoring therapy to individual needs. Another practical limitation lies in the need for the virtual avatar to be perceived as comparable to commercial gaming platforms. Experts suggested that high-quality design and activities, along with elements like “wrong feedbacks” that allow patients to self-correct, would enrich engagement, fostering a sense of accomplishment and autonomy.

Despite the promising use of robotics and gamification in supporting repetitive therapeutic movements, experts pointed to a limitation in the system's effectiveness for functional therapy tasks. Activities essential for daily living, such as dressing or pouring water, rely on proprioceptive learning that is best achieved with real-world objects. This gap highlights an area for improvement in extending the system's applicability to a broader spectrum of therapeutic needs.

Furthermore, the experts emphasized the critical differences in motivational needs between adults and kids. While adults may have intrinsic motivation to complete therapy exercises, kids, especially younger ones, may not. For kids, gamification and a compelling narrative could make therapy more engaging, but the experts also noted that the physical presence of a therapist might still be necessary in certain cases to ensure compliance. Systems where AI plays an important role, such as the one proposed in this paper, are indeed promising; however, kids represent a particularly complex challenge. When training an AI model to understand the state of a patient, it often relies on the patient's subjective self-evaluation to label the dataset effectively. In the case of cognitive disabilities, especially with kids, this ability is often lacking, and recent research suggests that the most viable approach is to base labeling on the opinion of a therapist who knows the patient well after years of working together–a solution that remains challenging to replicate in autonomous systems.

## 6 Conclusions and future works

In conclusion, our innovative AI-based system stands as a transformative approach in the realm of neurorehabilitation, offering a viable solution to the scarcity of specialized care professionals. By harnessing the capabilities of a socially interactive agent integrated within a robotic framework, we have successfully demonstrated the potential to replicate the critical social interaction and motivation factors found in traditional therapy settings. The system's flexibility allows promoting at-home rehabilitation with less dependency on professional availability. Qualitative feedback from participants underscored the user interface and the virtual coach's anthropomorphic attributes as pivotal in maintaining engagement, with users reporting a heightened sense of companionship and support that spurred consistent use. Additionally, insights gathered from interviews with neurorehabilitation experts provided valuable perspectives on both the system's strengths and limitations. The experts validated the potential of our approach, particularly for domestic rehabilitation, but emphasized the need for therapist oversight, adaptability in diverse home environments, and careful tuning of the avatar's behavior for different patient profiles. This feedback not only affirms the direction of our study but also offers a clear path for refining the system's design and addressing key challenges in future iterations. The encouraging outcomes of our feasibility study with healthy patients, showcasing their adaptability to the system and heightened engagement without distraction, lay the groundwork for further research. Looking forward, we intend to expand the scope of our research by conducting extensive trials with real patients suffering from neuromotor dysfunctions. These future studies will not only allow us to validate the efficacy of our framework in a clinical setting but will also enable us to perform a comparative analysis of rehabilitation outcomes with and without the presence of the interactive agent. This will offer a clearer understanding of the agent's impact on patient engagement and recovery. Additionally, to ensure the robustness and generalizability of our findings, we plan to increase our sample size, providing a more comprehensive understanding of the system's effectiveness across a diverse patient demographic. Through these endeavors, we aspire to refine and validate our system, making a significant contribution to the field of neurorehabilitation and providing a path toward more accessible and personalized patient care.

## Data Availability

The raw data supporting the conclusions of this article will be made available by the authors, without undue reservation.
